# Pre-School Teachers’ Knowledge, Belief, Identification Skills, and Self-Efficacy in Identifying Autism Spectrum Disorder (ASD): A Conceptual Framework to Identify Children with ASD

**DOI:** 10.3390/brainsci10030165

**Published:** 2020-03-13

**Authors:** Sahar Taresh, Nor Aniza Ahmad, Samsilah Roslan, Aini Marina Ma’rof, Sumaia Zaid

**Affiliations:** 1 Department of Foundations of Education, Faculty of Educational Studies, University Putra Malaysia, Serdang 43400, Malaysia; sahartaresh@yahoo.com (S.T.); samsilah@upm.edu.my (S.R.); ainimarina@upm.edu.my (A.M.M.); 2 Department of Psychology, Sana’a University, Sana’a 1247, Yemen; sumaiamohammed@hotmail.com

**Keywords:** preschool teachers, self-efficacy, knowledge, belief, skills, identify, autism spectrum disorder (ASD)

## Abstract

Recently, the identification and detection of children with autism spectrum disorder (ASD) has become an essential issue under ASD intervention services. The high percentage of ASD among children requires preschool teachers to recognizse children’s abnormal development and identify them at an early stage, followed by referral to specialists. Therefore, this identification calls for a specific ability among preschool teachers, identified as knowledge, belief, identification skills, and self-efficacy (KBISSE). This conceptual framework aims to utilize the current literature to present a discussion on preschool teachers’ KBISSE in identifying children with ASD and making decisions to refer children suspected with ASD to specialists. The conceptual framework is discussed based on social cognitive theory (SCT) and the health belief model (HBM). The conceptual framework emphasizes the need for preschool teachers to be educated in ASD via an educational module that could increase teachers’ self-efficacy in identifying children with ASD. Besides, knowledge in ASD, belief in ASD, and identification skills are also necessary variables for building the educational module. The educational module is useful for guiding future research on preschool teachers’ identification of children with any disability, one of which is ASD, and subsequent specialist referral at an early stage.

## 1. Introduction

Recently, professional concern towards children with behavioral difficulties is now viewed as an integral part of the teacher’s role [1]. One of these behavioral difficulties is an autism spectrum disorder (ASD).

ASD is considered the most common multifactorial disorder affecting children today [2,3,4]. Currently, the high percentage of ASD among children requires preschool teachers to recognize children’s abnormal development and identify them at an early stage followed by referral to specialists [5,6,7]. A preschool teacher has a high chance of detecting this type of disorder among his or her students and could identify the student’s situation to refer them for appropriate assessment towards obtaining early intervention services [8]. However, preschool teachers might assume many obstacles in identifying and referring their students for assessment [8,9,10].

These obstacles could include personal characteristics of preschool teachers such as the preschool teachers’ beliefs, attitudes, feelings, skills, and perceptions of children with ASD, and the preschool teachers’ knowledge in managing ASD. So, the lack of knowledge coupled with incorrect beliefs toward ASD could lead to preschool teachers having a weak self-efficacy in identifying ASD in children and less confidence in voicing their concern to the children’s parents and then referring them for early intervention. 

Self-efficacy is one of the teachers’ characteristics that reliably affects their teaching practices, classroom teaching, and communication with children [11]. According to Bandura [12], self-efficacy can be defined as the preschool teacher’s ability to take action and handle children with challenging behavioral problems like ASD (p. 270). One study suggested that teachers who believed in their ability to handle behavioral issues like ASD would put in the effort to create change for the affected children, and vice versa [13], because preschool teachers have the most important role in identifying children with ASD and referring them for clinical intervention at an early stage [8]. 

An important party in the early identification of children with ASD is preschool teachers, as they are considered reliable resources for intervention issues [14,15,16] due to their role in dealing with parents to point them towards intervention services. Furthermore, preschool teachers deal with children daily and have been educated in child development [17,18]. Due to these specific characteristics, preschool teachers should have the best qualities to identify children who do not exhibit signs of normal development at an early age [9]. 

Other researchers have found that a shortage of preschool teachers’ knowledge and skills in handling behavioral difficulties was the main factor affecting their referral ability [19]. Another study found several teachers with a shortage of knowledge and skills in handling preschoolers with challenging behavior and recommended them for training [20]. 

In other words, the preschool teachers’ beliefs, understanding, knowledge, and skills related to preschoolers’ challenging behaviors might impact their identification of ASD and referral decisions later [14,15,16,19]. 

**Figure 1:**
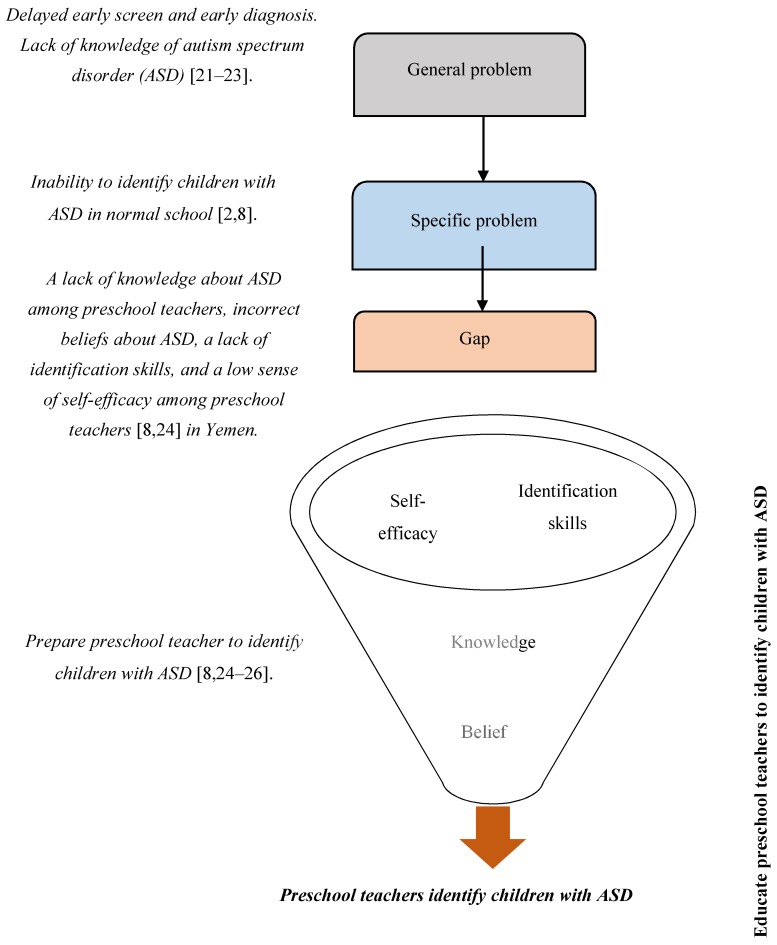
The research problem statement [2,8,21,22,23,24,25,26].

This study attempts to explore how preschool teachers can acquire the ability to identify children with autism and refer those suspected with ASD to specialists while working with the children’s parents at the same time. Furthermore, the present research studies the effect of preschool teachers’ ASD knowledge and skills in identifying children with ASD. It aims to correct the misbelief in society regarding ASD, and to increase preschool teachers’ self-efficacy in recognizing the symptoms of ASD, the factors to increase their confidence to voice their concerns to parents, and boost their willingness to refer children for screenings and other services. Therefore, the main purpose of this study is to conduct a comprehensive discussion of the literature and existing theories to build a conceptual framework that would prepare preschool teachers to identify children with ASD and make the decision to refer children suspected with ASD to intervention services. Figure 1 shows the problem statement identified in this study.

### 1.1. Autism Spectrum Disorcer (ASD) Knowledge among Preschool Teachers 

ASD knowledge among preschool teachers refers to general information on ASD, symptoms of ASD, ASD treatment, and the etiology of ASD among preschool children. Thus, the teachers’ knowledge affects their identification of children with ASD and their referral decisions [14,15,16,19].

The preschool teachers’ lack of knowledge in screening and identifying children with ASD becomes one of the most significant barriers in the intervention issues of ASD [21]. As defined by several studies around the world, there is a lack of ASD knowledge among teachers [22,23,27] particularly regarding the early signs of ASD [28,29,30].

The *Diagnostic and Statistical Manual of Mental Disorders* (*DSM*) 2013, has classified or categorised many disorders under ASD such as Asperger disorder and pervasive developmental disorder. Furthermore, they have determined just two main criteria for ASD diagnosis. One is difficulties in social communication and the other is restrictive and repetitive patterns of behaviour. These new classifications and criteria shifted the way people think about autism and enhanced the development of many instruments related to diagnosis tools of ASD, people’s knowledge about ASD, and their ability to identify ASD [31]. However, in developing countries like Yemen, it is a big challenge to use some of these ASD diagnosis tools for several reasons [31,32]. First, using these tools needs well-trained clinicians and experts to ensure the accurateness of the diagnosis [32]. Second, the lack of centers and experts who work with autism. Moreover, Yemeni preschool teachers don’t have in-depth knowledge about ASD and are not trained to deal with special needs students in general [33,34]. Therefore, they might not be qualified to use ASD diagnosis tools to avoid wrong interpretation of the outcomes of these tools. Besides, studies have indicated that only 8% of assessments of ASD among children use a formal measure while most use informal assessments [32,33].

Hence, recent studies are now insisting on educating teachers about the early signs of ASD to enable them to identify early symptoms of ASD and to refer the children to professional assistance in the first stage of childhood [22]. Preschool teachers with a low level of ASD knowledge require urgent training [35,36].

Past studies on teachers found that an absence of knowledge and skills in handling early childhood students with challenging behaviors is an obstacle in the detection and intervention of ASD. The literature shows that preschool teachers have been questioned about the factors they observed as impacting their referral decision of children with behavioral difficulties—to which they expressed a lack of knowledge as one of the most crucial factors affecting this ability [9]. Moreover, the teachers rated the identification of ASD and the referral of suspected children with ASD as more important than any other issue [20]. (For more details of the literature review in the knowledge of ASD, see Table 1). 

According to Bandura [44], even if the individuals have the knowledge to complete a task, it does not guarantee that they would actually implement the task. The research mentioned the central role of self-efficacy in gaining the knowledge to be applied in one’s work and what one actually does. Self-efficacy is usually deliberately discussed with knowledge because, as some researchers have figured out, base-level knowledge may be needed to be able to perform some actions [45]. This relationship between knowledge and self-efficacy directly influences the individual’s performance or capabilities in an instructive system. As teachers, self-efficacy tends to be the most crucial role in impacting their confidence in implementing their knowledge in several situations [46], as cited in Soto et al. [47].

In contrast, other studies found that the strongest factor contributing to high self-efficacy was “confidence in knowledge” (e.g., obtained via teaching experience, teacher training, professional development, and personal knowledge). When asking 84 pre-service teachers and 156 experienced teachers to name the factors that affect increased self-efficacy [2], the most frequently cited was confidence in knowledge for experienced teachers; but the most popular reason for high self-efficacy among teachers was cited to be personal qualities (e.g., concerned attitude, motivation, positive position, and the ability to get along with people) [48]. That is, both groups cited confidence in knowledge, teaching experience, and managing the class as the factors that most influenced self-efficacy. Therefore, knowledge is essential in gaining self-efficacy. Besides, this result mirrors Bandura’s [44,49] view that recognized knowledge and experience as a form of behavioral capability and the main cause of self-efficacy.

According to the above association between knowledge and self-efficacy, another crucial variable to consider is the assessment of the preschool teachers’ level of knowledge in dealing with abnormal development such as ASD. Specifically, several works have indicated that, generally, teachers do not have the capabilities necessary to deal with special-needs children [50]. Therefore, some noted that although there is a larger than average need for special-needs education services such as a head-start program, there are insufficient resources (such as trained personnel) to address these needs [16].

### 1.2. Beliefs about ASD among Pre-School Teachers

Beliefs “play a critical role in defining behavior and organizing knowledge and information” (p. 328) [51]. The health belief model (HBM) suggests that a person’s belief regarding a personal threat of an illness or disease, together with a person’s belief regarding the effectiveness of the recommended health behavior or action, will predict the likelihood that the person will adopt the behavior.

Other investigations found that there are valid causes for realizing the educational beliefs of pre-service teachers as essential to the teachers’ education module—because these beliefs majorly impact the pre-service teachers’ knowledge achievement, their clarification of knowledge and course content, teaching behavior, task description and collection, and “comprehension monitoring” [51] (p. 313–328) [52]. Pajares [53] defined that beliefs reflect some type of understood knowledge. Also, the author determined that some scholars view beliefs as a portion of knowledge, while others view beliefs to be a portion of conception. Furthermore, the author declared that these beliefs could shape “one part of an individual’s meta-cognition” (p. 2). One study proposed an interesting assumption that “beliefs influence what teachers say outside the classroom, but their behavior in the classroom is a result of beliefs measured and filtered by experience. Also, their knowledge represents their efforts to make sense of their experience” (p. 312) [51].

The belief regarding ASD refers to teachers’ emotional state and concerns about the children’s behavior that influences their ability to identify, voice their concern to the children’s parents, and then refer them to specialists and time their decisions.

This belief is divided into two types: religious and personal beliefs. Teachers’ belief reflects the difficulty in voicing or discussing their concerns about their perception of the difficult behavior of the child to his or her parents, even addressing or referring the preschooler as having challenging behavior based on the misbelief surrounding ASD among teachers and parents [9]. Furthermore, other concerns related to a person’s beliefs can affect the central role of how individuals understand and explain incapacity and children with ASD [54].

Studies have indicated that feelings may also impact teachers’ identification and referral decisions. For example, teachers may view it as easier to talk with parents regarding their children’s speech and language problem rather than discussing the possibility of a mental disorder, as the former is less stigmatizing than the latter. Fantuzzo et al. [14] agreed that identifying children with speech and language problems even when there is no speech or language problem present may be done as a means to “avoid the negative consequences of a more stigmatizing and continuing label” (p. 478). This view suggests that there may be biases or fears related to mental health or illness in general and in early childhood in specific. Besides, Fantuzzo et al. [9] proposed that teachers might experience stress when dealing with a child with behavioral problems such as hyperactivity, causing a social disturbance, consideration problems, and non-subjugation. Making a referral to speech and language services, because they are more accessible and less stigmatizing, may provide teachers with more direct help than what may be obtained by waiting for mental health services. While all these suggestions appear sensible, an additional study on teachers’ beliefs that cause them to harbour bias against making referrals because of feelings and behavioral problems has yet to be conducted.

In this study, preschool teachers’ belief was divided into two: religious and general. Religious belief refers to religion and spiritual traditions that are often associated with health practices observed in cultures around the world [55]. Teachers’ beliefs affect their ability in making referral decisions, as Muslim families believe that God puts an autistic child under their care not only because of fate or reincarnation but also because God wants to assess the families to see if they could take care of the child [54]. Religious implications on the beliefs about children with developmental problems are not only limited to Muslims believers alone [3] but are also inclusive of other religious groups. For example, as reported in a previous study, 55% of Latina mothers believed that their autistic child is a sign of God’s existence [44], such as, Latina mothers believe that ASD is blessings or gifts from God. [45]. Latin Americans have the option of opting for non-traditional treatments, and numerous Hindu parents of children with “mental disorders” believe that God has given them the child as a response to sins committed in their previous lives [47]; also, Americans use traditional treatments and professional services with behavioral health [46], and ultra-orthodox Jewish families often change community dynamics by receiving medical advice from a Rabbi [48].

Meanwhile, general belief refers to culture and personal belief. This belief is related to society’s common belief system and serves as an explanatory model for disorders such as ASD. On the negative side, culture makes people perceive ASD as a stigma. The stigma surrounding autism has resulted in discrimination not only against autistic children but also their families [56]. Moreover, most children with ASD have gone unidentified due to the fear of social opinion among the parents and children [55]. Furthermore, the *Diagnostic and Statistical Manual of Mental Disorders*, Fifth Edition (*DSM-5*) considers culture as the main standard for judging whether or not certain behavior is abnormal. Several studies have focused on the role of culture, society, race, and the types of social relationship factors in determining beliefs regarding psychological disorders.

Moreover, personal belief is related to preschool teachers’ diagnosis of ASD causes and symptoms, or general information that reflects their attitude or thinking [51]. Besides, this belief often differs from groups of people such as those with low education level, or some individuals with unique characteristics, like those of the Arab community. For example, the common Arabic word for autism describes individuals with a behavioral, mental, physical, and emotional disability, but often the term is translated to ‘introvert’ or ‘withdrawn’ in English or (التوحد) in Arabic. Hence why many individuals may incorrectly describe the nature of ASD as introversion [57]. Furthermore, the literal translation of both Chinese terms for autism (GuduZheng or ZibiZheng) is similar to ‘loneliness’ or ‘introvert disease,’ which implies a more psychological etiology [58]. (For more details about the literature review in beliefs of ASD, see Table 2).

Theoretically, the health belief model (HBM) can explain preschool teachers’ beliefs, as this theory describes health-related behaviors and medical decision-making. The HBM was initially developed in the 1950s to explain why people did not participate in preventive disease programs [68,69]. Preschool teachers’ beliefs can be a barrier preventing them from performing health behaviors for the children. Besides, the model could also explain how preschool teachers’ beliefs affect their actions to protect children in the class and help them take the appropriate action [70].

The judgments of preschool teachers regarding the perceived barriers and the perceived benefits of an action define the course of the action taken; these two components together form the dimension of outcome expectations [70]. Preschool teachers’ “perceptions of the costs involved in seeking a diagnosis” include time, not having evidence, social stigma, how to voice their concern to parents, not knowing who to contact, refusal of the parents, etc.

Moreover, some researchers have described the difficult behaviors as challenging, and these behaviors could lead to a delay in referral decisions over a more extended period. In fact, several teachers said that they were unwilling to allocate a stigmatizing label to the children and worried about the parents’ reactions to their valuation of their child’s problematic behavior. All of these pertain to the component addressed as perceived barriers [2,9]. As several studies have confirmed, ASD beliefs affect teachers’ decision-making, finding that special education teachers agreed with common features and misconceptions of autism more than authentic reports of autism specialists [71]. The study also assessed teachers’ and parents’ belief and knowledge related to various aspects of ASD, finding that both groups had misbeliefs related to cognitive, developmental, and emotional aspects of ASD [71]. Both the teachers and the parents believed that children with ASD were mentally delayed but more often agreed that the children had special talents and were more intelligent than test detections [71]. Moreover, these misbeliefs could result in an overly high tendency for schools and homes to interpret the disorder as “stubbornness” instead of deficits in understanding or ability [71]. This misbelief is attributed to teachers’ overestimation of children during diagnosis [72], becoming barriers that prevent teachers’ from taking appropriate action.

These barriers of beliefs are defined as detrimental to self-efficacy in taking action, seeking diagnosis, and in making referral decisions [8] due to insufficient training, conflicts with specialists on interference, and a lack of referral plans [73].

Therefore, teachers must have accurate knowledge and beliefs about autism to meet the complex behavioral needs of children with autism. This situation is especially important, as some of the exceptional skills of students with autism may cause teachers to misinterpret students’ social and learning skills, and consequently, provide insufficient support [74]. This issue could be addressed by providing the teachers with appropriate education and training.

### 1.3. ASD Identification Skills among Pre-School Teachers

Skills are known as the “ability to do something well; in other words, it is the ability to use one’s knowledge effectively and readily in behavioral execution or performance” [75]. In this study, skills are referred to as the preschool teacher’s skills to identify children with ASD by implementing their knowledge regarding the risk and symptoms of ASD.

Most people perform early screening and identification of ASD utilizing the knowledge and skills of early childhood specialists or through persons with daily constant contact with the child such as preschool teachers [76]. However, several factors act as barriers to preschool teachers’ identification or decision-making and then referral of the child for early intervention [9]. One of these barriers include skill [14,16,19,20,77,78]. There is a specific skill to elicit and recognize early markers of ASD [36]. Thus, preschool teachers need to improve on skills in observing growing children, recording their behavior, and lastly writing a report.

Furthermore, these skills need to be determined to give teachers the necessary skills to influence parents, collaborate with them, and negotiate an appropriate referral system [9]. Some studies have confirmed that preschool teachers’ lack of knowledge and skills have impacted the management and determination of behavioral problems among abnormal children with ASD [19,20]. Therefore, several preschool teachers have expressed the need for more training to handle challenging behaviors such as ASD [20,79].

This study aims to provide preschool teachers with important identification skills to help them identify children suspected with ASD. These identification skills are observation skills, recording skills, and report-writing skills (Table 3).

Observation is the process of looking at a child at work or play without coming off as nosy. It is considered an essential tool for acquiring information, gaining results, and generating ideas. Recording is defined as one of the observation skills involving writing down an observed activity or behavior. Although several teachers are used to recording, a regular method will guarantee that the children are properly observed while participating in many different activities at a time. With these steps, preschool teachers will be able to describe the child’s behavior and write down important information. 

In the social cognitive theory, Pajares [51] confirmed that self-efficacy cannot lead to requisite behavior when skills and knowledge are lacking. Instead, “competent functioning requires harmony between self-beliefs on the one hand and the possession of skills and knowledge on the other” (p. 3) [53]. To foster competent functioning development among preschool teachers, the teachers must have correct understanding, knowledge, and skills regarding ASD, especially given their critical role in supporting effective teaching and referral [84,85]. To assess self-efficacy, a person’s specific skill sets or particular skills related to particular challenges or topics must be evaluated. Hence, when preschool teachers are exposed to education or training programmes on knowledge and skills, the most significant outcome will be increased self-efficacy [36]. Drusch [8] confirmed that professional or personal experience is not enough to increase a teacher’s knowledge and skills. In a similar vein, teachers cannot gain knowledge and skills in identifying and recognizing ASD just by working with children. Instead, the teachers need to be exposed to more training and equip themselves with the skills to recognize ASD symptoms among children. In doing so, they will increase their ability to refer these children to the appropriate services.

### 1.4. ASD Self-Efficacy among Preschool Teachers

Self-efficacy is one of several teacher-related characteristics that is consistent with effective teaching practices, classroom learning, and communication with children [11]. According to Bandura’s [86] theory of social cognition, self-efficacy refers to an individual’s belief (i.e., one’s confidence in one’s competency to do a particular task) and has the most powerful effect on the behavior and motivation of the individual. However, this power is linked to other variables such as knowledge, skills, beliefs, attitude, and the individual’s intention [44]. Based on Bandura’s [60] definition, self-efficacy refers to preschool teachers’ beliefs about their ability to successfully perform a particular behavior [36,87,88]. Moreover, a high sense of self-efficacy is positively related to promising results. For example, it is associated with encouraging in-class behavior, classroom practice, use of praise more often than criticism, increased perseverance with “low achievers,” spending more time monitoring student performance, and spending more time on class preparation and paperwork, increased willingness to collaborate with other professionals regarding student concerns, increased significant effort, and increased success [87,88,89,90,91].

Several researchers claim that high-level knowledge among teachers could correlate to increased self-efficacy. For instance, Sasson [36] found a significant correlation between knowledge and self-efficacy among allied health professionals. The study noted that increased knowledge in ASD could increase the clinical confidence of health professionals. Bandura [60] confirmed these findings, stating that both knowledge and performance are thought to affect self-efficacy [44]. Therefore, studies have turned to teacher training to explain the gap in study and practice. Moreover, based on Bandura’s [60] theory, teachers who believe in their ability to address behavioral problems such as ASD would work towards making a difference for those children (p. 560). Hence, self-efficacy is one of the most crucial factors affecting whether or not the teachers will apply classroom-based training programs or educational modules in early childhood development and whether or not they would be able to identify problematic behavior such as ASD [92].

As it is known, ASD is a childhood development disorder (CDC, 2013) [93]. Hawley and Williford [94] identified children with ASD as requiring teachers with high self-efficacy so that the likelihood that these children are transferred to intervention services is increased [94]. Furthermore, a high sense of self-efficacy among preschool teachers would cause them to recognize children with ASD as a complex problem. However, teachers who lack the knowledge to recognize the behavioral problems of ASD [8] will not have high-level self-efficacy and would not be confident in recognizing children with ASD [36]. For more details about the literature review in self-efficacy in ASD, see Table 4. 

Numerous evidence has confirmed the association between one’s self-efficacy and confidence in one’s ability to carry out a task [8,9,36]. However, some studies found teachers to be generally confident in their ability to deal with children with ASD, while others found that teachers had a low level of confidence regarding special-needs children suggesting they need more training in special education [97]. In line with Bandura’s [60] theory, a teacher with more knowledge and training specific to catering to children with ASD would have higher self-efficacy to deal with the affected children and could identify them early on [9].

In this study, preschool teachers are defined as having two types of self-efficacy: (1) The ability to discuss with parents and counselors, and (2) the confidence to help the diagnostic team.

### 1.5. Theoretical Rationale

The conceptual framework developed in this study was based on two theories: Bandura’s [60] social cognitive theory (SCT) and Rosenstock et al.’s [70] health belief model (HBM).

#### 1.5.1. Social Cognitive Theory (SCT)

This theory was initially called social learning theory (SLT) when introduced in the 1960s by Albert Bandura. Later, the theory was renamed social cognitive theory in 1986; positing that learning occurs in a social context with a dynamic and reciprocal interaction between the person, environment, and behavior.

One unique feature of SCT is its emphasis on social influence and external and internal social reinforcements. SCT considers the unique way in which individuals acquire and maintain behavior, while also considering the social environment in which individuals perform the behavior. The theory takes into account a person’s past experiences, which factor into whether behavioral action will occur. These past experiences influence reinforcements, expectations, and expectancies, all of which shape whether or not a person will engage in specific behavior and the reasons why a person engages in that behavior [98].

Many theories of behavior used in health promotion do not consider the maintenance of the behavior, but rather focus on initiating the behavior. This is unfortunate, as the maintenance of the behavior, and not just the initiation of the behavior, is the true goal of public health. The goal of SCT is to explain how people regulate their behavior through control and reinforcement to achieve goal-directed behavior that can be maintained over time. The theory provides a framework for understanding how people actively shape and are shaped by their environment. In particular, the theory details the processes of observational learning and modeling, and the influence of self-efficacy on the production of behavior [99].

Initially, Bandura [60] developed five constructs. Later on, the self-efficacy construct was added when the theory evolved into SCT. These constructs and how they relate to this study are explained in detail below:

1. Reciprocal determinism—this is the central concept of SCT that refers to the dynamic and reciprocal interaction of a person (in this case, preschool teachers with a set of learned experiences, level of education), environment (external social context), and behavior (responses to stimuli to identify children with ASD).
2. Behavioral capability—this refers to a preschool teacher’s actual ability to perform a particular behavior (to identify children with ASD) through essential knowledge and skills. To successfully perform the behavior, preschool teachers must know what to do and how to do it. Preschool teachers learn from the consequences of their behavior, which also affects the environment (class) in which they work.
3. Observational learning—this asserts that preschool teachers can witness and observe behavior conducted by others, and then reproduce those actions. This is often exhibited through the "modeling" of behaviors. If preschool teachers see the successful demonstration of a certain behavior, they can also complete the behavior successfully.
4. Reinforcements—this refers to the internal or external responses to a preschool teacher’s behavior that affect the likelihood of continuing or discontinuing the behavior. Reinforcements can be self-initiated or originate from the environment, and reinforcements can be positive or negative. This is the construct of SCT that most closely ties into the reciprocal relationship between behavior and environment.
5. Expectations—this refers to the anticipated consequences of a preschool teacher’s behavior. Outcome expectations can either benefit or not. Preschool teachers anticipate the consequences of their actions before engaging in certain behavior, and these anticipated consequences could influence the successful completion of the behavior. Expectations derive largely from previous experience. While expectancies are also derived from previous experience, expectancies focus on the value that is placed on the outcome and is subjective to the individual.
6. Self-efficacy—this refers to the level of preschool teachers’ confidence in their ability to successfully perform a certain behavior. Self-efficacy is unique to SCT although other theories have added this construct at later dates, such as the theory of planned behavior. Self-efficacy is influenced by preschool teachers’ specific capabilities and other individual factors, as well as environmental factors (barriers and facilitators).

#### 1.5.2. Health Belief Model (HBM)

HBM is a theoretical study that describes health behavior and medical decision-making skills. The original HBM was established in the 1950s. The model focuses on the behavior of individuals who have declined to participate in preventive disease programs [68,70]. The model has been implemented in several works aiming to study patient behaviors like dieting in obese children, factors of protection from skin cancer, and parenting skills programs to enroll associated parents with parental motivation [100,101,102]. However, HBM usually uses an individual’s health behavior. In this study, HBM was used to describe preschool teachers’ beliefs and how their actions predict the voicing of their concerns about children’s health [103]. HBM contains six components under four factors (threat exception-outcome, exception-self efficacy, exception-cues to action) [70]. Perceived barriers are one of the concepts under outcome exception. It refers to the barriers that prevent the preschool teachers from taking a particular action or voicing out his or her concern to the suspected child’s parents. These barriers include social stigma, not knowing who to contact to refer to, waitlists, etc. The perceived barriers held by preschool teachers can impede their identification of ASD and subsequently their referral decision [2,9]. An example of the preschool teachers’ barrier is that they believe ASD among children is normal and that children’s behavior will change as they mature [19].

Moreover, family culture and language differences are also considered as barriers that influence teachers’ ability to make a referral for a child with ASD [14]. Another source of reluctance in making referrals or discussions with parents is teachers’ negative perceptions and concerns about the parents, in turn, influencing the teachers’ ability as well. Several teachers are uncomfortable expressing their concerns that a child has ASD to a child’s parents because of parents’ reactions, as some parents attribute a stigmatizing label to ASD. Another study confirmed that teachers found it easier to tell parents that their children had verbal and language problems rather than a mental disorder, as the former is less stigmatizing and would have less potentially negative repercussions [104]. Ultimately, teachers’ concerns have a direct effect on their ability to take action on behalf of children with ASD. Concerns about labeling and communicating with parents may be closely related [14].

In the end, preschool teachers’ self-efficacy may play a role in their identifying and making decisions to proceed, as they pursue answers about their concerns for the children’s development to ultimately obtain an early diagnosis of ASD. Some studies have identified factors relating to teachers’ knowledge, observation skills, and their belief about young children with ASD that could influence their decisions to make referrals [14,15].

## 2. Methodology

The present study reviewed the literature and theories in-depth to look for evidence of factors that could improve preschool teachers’ ability to identify children with ASD. The study focused on some variables to enhance teachers’ knowledge and to change their beliefs regarding ASD overall and the early signs of ASD specifically, and to equip them with identification skills in ASD, besides enhancing their self-efficacy in identifying children with ASD.

## 3. Result

According to an in-depth literature review, preparation of preschool teachers becomes an essential step to support early diagnosis through early identification in preschool. Preparation of preschool teachers should be done by building an educational module. Therefore, according to the results of the present study, it is evident that several elements can be used to prepare preschool teachers to identify children with ASD. These elements include knowledge and beliefs, identification skills, and self-efficacy, which this study conceptualized as essential elements for the proposed framework, with suggestions for an educational module besides experimental testing as a valuable contribution to the literature.

### Conceptual Framework Development

**Figure 2:**
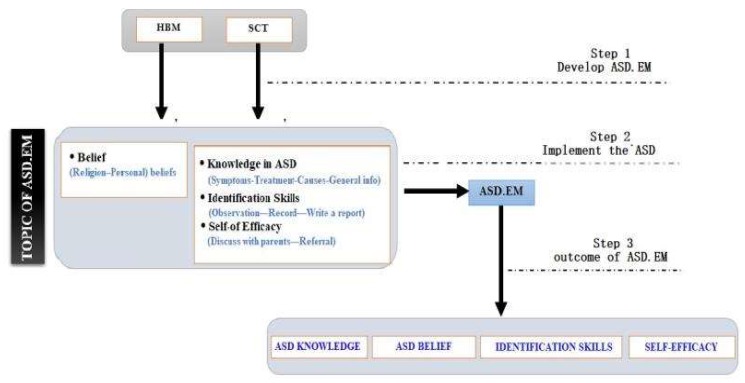
A conceptual framework for identifying children with autism spectrum disorder (ASD).

The conceptual framework aims to prepare preschool teachers to identify children with ASD. The conceptual framework contains several variables, namely knowledge in ASD; identification skills in ASD; belief in ASD; and self-efficacy in identifying children with ASD. Some variables have several sub-variables, as shown in Figure 2.

One of the barriers to ASD identification is preschool teachers’ lack of knowledge in the area of ASD [8,21,105]. Knowledge of ASD here refers to preschool teachers’ knowledge and information about ASD, symptoms of ASD, its causes and treatment. Preschool teachers could be exposed to these variables via an educational module. Based on SCT, increased knowledge among preschool teachers regarding ASD signs will increase their self-efficacy in identifying children with ASD [36,97,104].

**Figure 3:**
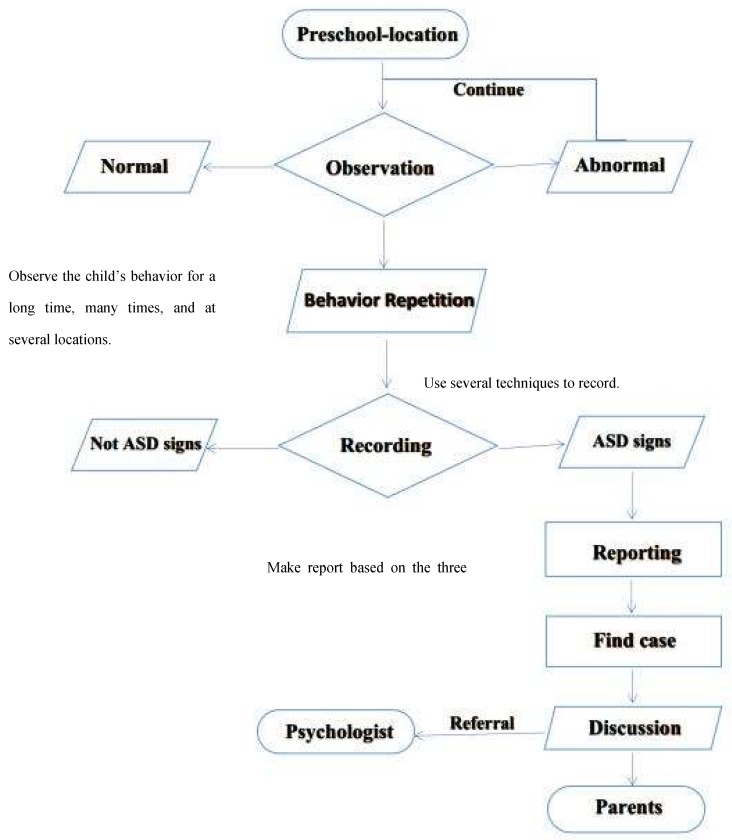
Identification skills process.

Secondly, identification skills refer to a particular part of this conceptual framework relating to the identification skills that preschool teachers needed to improve upon. These skills are observation, recording, and reporting (see Figure 3).

As shown in Figure 3, in the preschool location, teachers can exercise their identification skills by following the steps below:

The preschool teacher observes the children under her care under several situations in class, outdoors, during teaching activities, and/or playing activities. In case a child exhibits abnormal behaviour, the preschool teachers move to the next step of recording the behaviour in different locations using different technical skills. The preschool teachers should be looking for ASD warning signs in communication and social interaction, and patterns of behaviour, and interests in their activities. If the preschool teacher observes the behaviour repeated many times, she should record her observation and move to the third step, which is to write a report [106]. After the preschool teacher determines the suspected child, she should share her concerns on the atypical developments with a psychologist or the child’s parents or both and show them the reported behaviour and ask them to refer the child to a specialist if necessary [107].

Thirdly, the belief variable refers to teachers’ feelings and concerns regarding children’s behaviour that influence their identification and voicing out concerns to children’s parents, their referral decisions, and the timing of their decision. This belief is divided into three categories (religious, societal, and personal). These variables are considered the third barrier preventing preschool teachers from identifying ASD. Also, the hypothesis underlying these variables states that if inaccurate beliefs among preschool teachers are reduced, their ability to identify ASD will increase and they will be more confident to voice their concerns with children’s parents.

Finally, self-efficacy in identifying children with ASD is a variable that refers to preschool teachers’ ability to identify ASD symptoms, discuss with parents, and the confidence to make referral decisions [8]. The main goal of this framework is to enhance preschool teachers’ ability to identify children with ASD in preschool. Therefore, a hypothesis is proposed that if preschool teachers have a high level of preparation in identifying children with ASD, their ability to determine the red flags indicating ASD among children will increase. In turn, teachers will improve their ability to voice their concerns to the child’s parents, and ultimately, they can make the decision to refer the child for formal diagnosis and then for early intervention.

## 4. Discussion

This study is a concept paper, that presents a discussion on preschool teachers’ ability to identify children with ASD via a review of past studies and existing theories to develop a conceptual framework. This conceptual framework helps preschool teachers prepare to identify children with ASD based on different variables such as knowledge in ASD, belief in ASD, identification skills, and preschool teachers’ self-efficacy in identifying children with ASD.

This study aimed to determine the association between knowledge and identification of children with ASD. Preschool teachers’ knowledge in ASD is considered the most important factor that helps them identify early signs of ASD. Preschool teachers cannot take action without having basic information about the disorder [50]. Besides, preschool teachers must be educated in the early signs of ASD to be able to deal with this kind of behavioral disorder [16]. Also, the barriers preventing teachers from detecting ASD at an early stage should be removed.

On the other hand, this study discussed other barriers preventing preschool teachers from identifying children with ASD, one of which is preschool teachers’ beliefs. These beliefs are identified as barriers preventing them from identifying children with ASD. Preschool teachers cannot take action or refer the parents of the child suspected with ASD to specialists because some parents still perceive ASD as a stigma. Moreover, some preschool teachers still have misbeliefs and often attribute this disorder to “stubbornness” instead of deficits in mental ability [71]. As per the health belief model (HBM) incorporated in the conceptual framework of this study, these barriers among preschool teachers could be reduced, as several studies have confirmed [2].

Also, preschool teachers’ competent functioning development must be aligned with correct understanding, knowledge, and skills, especially given the critical referral role that they have [84,85]. The specific skill sets or particular skills related to particular challenges or topics such as teachers’ identification of children with ASD should be evaluated. This study proposed examining teachers’ self-efficacy to explain their ability to do so [36].

Preschool teachers must have the ability to refer children suspected with ASD to specialists and discuss the issue with children’s parents. Therefore, they must have knowledge of early signs of ASD, correct belief, and ASD identification skills. Preschool teachers must have the self-efficacy to identify children with ASD. However, more than a few studies have mentioned insufficient resources such as training programmes or educational modules to educate preschool teachers in identifying children with ASD [16]. So, this study suggested using an educational module to address these challenges. Also, the study framework will help improve preschool teachers’ ability to identify children with ASD, as confirmed by several studies [36,59].

## 5. Conclusions

This study offered a specific emphasis on the early identification of children with ASD, which will further improve early diagnosis and early intervention for the children. Furthermore, the lack of knowledge and incorrect beliefs among preschool teachers and parents should be closely examined to increase the percentage of children that obtain correct diagnoses within the appropriate time, as this would help to significantly impact the children’s actual behavior.

It is important to highlight the limitations of this study. Firstly, the approach of this study was to build a conceptual framework that may not be backed by experimental work. Secondly, other factors may affect preschool teachers’ ability to identify ASD but were not discussed in this study, such as experience working with ASD, intention, and attitude. Thus, this study calls for a more qualitative and quantitative approach to assess the factors that can increase preschool teachers’ self-efficacy in making decisions and referring children with ASD for diagnosis. Moreover, the benefit of the proposed module in the long term was not examined in this study. Moreover, this study did not focus on parents with ASD children. Therefore, future works should focus on preparing parents in identifying whether or not their children have ASD to support early detection and early intervention at an early stage.

## Figures and Tables

**Table 1:** Summary studies of knowledge about autism spectrum disorder (ASD).

Authors\years	Objective	Sample	Instrument	Result	Conclusion
Taresh et al., 2020 [33]	The current study aimed to figure out what is the pre-school teachers’ knowledge about ASD. Besides, this study attempted to find out if there are any significant differences in preschool teachers’ knowledge about ASD in relation to their education level and teaching experience.	A total of 300 preschool teachers from various region schools in Taiz City in Yemen.	Questionnaire to determine their level of knowledge about autism.	The results indicated that preschool teachers had a lower level of knowledge about the disorder. The findings also showed significant differences in the teachers’ knowledge about autism, depending on their education level and teaching experience.	In conclusion, Yemeni preschool teachers need more education and training in autism spectrum disorder.
(Hof M., 2020) [1]	The study evaluated the knowledge of ASD and stigmatizing attitudes.	Physicians at Dutch Youth and Family Centers (YFC).	Questionnaire.	The physicians had positive attitudes toward mental illness but they had higher levels of stigmatizing attitudes than other Western healthcare professionals. Their levels were considerably lower than in non-Western professionals. We found no relations between ASD knowledge, stigmatizing attitudes, and demographic variables.	In conclusion, ASD knowledge and stigmatizing attitudes toward mental illness in Dutch YFC physicians require attention.
(Badam, 2019) [37]	The current study aimed to determine and compare the awareness level of ASD among participants including medical and non-medical professionals.	The participants were nursing trainees versus teachers(200 participants), 100 nursing trainees and 100 teachers.	A questionnaire comprising of 19 questions categorized in five sections on various communication disorders.	The current study found a higher level of awareness of communication disorders amongst the medical professional and non-medical group.	It concluded that there is a need to spread constant awareness by awareness campaigns about ASD.
(Sasson, 2018) [35]	To examine the effect of an early screening training on pediatric Physical Therapists (PTs): (1) Knowledge of autism spectrum disorder (ASD), (2) clinical self-efficacy, and (3) identification of markers.	Twenty-six pediatric PTs participated in a two-day ’Early ASD Screening’ workshop.	Questionnaire in both ASD knowledge and self-efficacy, and video case study	The result confirmed that there is an increase in PTs’ knowledge and self-efficacy after the ASD workshop, as compared to before the workshop, and the PTs’ ability to identify the early signs of ASD is greater than before the workshop.	It concluded that the workshop was useful to increase the level of knowledge and self-efficacy among PTs.
(Rakap et al., 2018) [38]	They examined the teachers’ knowledge and perceptions of ASD.	A total of 478 general education teachers in Turkish schools.	Questionnaire in knowledge and self-efficacy.	The teachers have a low level of knowledge and misconceptions about ASD.	The results confirmed that there is an urgent need to develop module or certification programs to train teachers to understand this kind of disorder and to work with ASD childrens’ implications for future research.
(Sanz-Cervera et al., 2017) [39]	This study aimed to examine and compare the pre-service teachers’ knowledge, misconceptions, and gaps about autism in their first and final year at university.	Pre-service teachers, n = 866.	Questionnaire.	The finding showed that fourth-year students had higher levels of knowledge and fewer gaps than the first-year students, although they also had more misconceptions. Special education specialists obtained significantly more knowledge and fewer misunderstandings than the general education pre-service teachers. However, specific training and experience had a significant influence on the knowledge and gaps, but it had no impact on the number of misconceptions.	These results suggest that university preparation in autism spectrum disorder (ASD) might not adequately train all future teachers.
(Heys et al., 2017) [40]	Examined parents’ and professionals’ understanding of autism in one low-income country, Nepal.	Parents of autistic and non-autistic children and education and health professionals, n = 106.	Semi-structured interviews.	The result showed there was a lack of knowledge among the participants. This study shows the striking lack of awareness of autism by parents and professionals alike in one low-income country.	
(Al-Sharbati et al., 2015) [24]	Studied children with special needs such as those with an autism spectrum disorder.	A total of 164 teachers were randomly selected through five schools.	A cross-sectional study to gauge the knowledge and attitude of mainstream school teachers towards ASD in an urban region in Oman.	The results confirmed that misconceptions about autism spectrum disorder were found to be common among mainstream teachers in Oman.	
(Shamsudin, Rahman Abdul, 2014) [41]	The study aimed to provide preliminary insight into the awareness of children with autism among the general public in Malaysia.	The general public in Malaysia, n = 250.	Questionnaire.	This study found that, although there are many Malaysians familiar with the term autism, most of them still do not really understand the characteristics of children with the disorder.	
(Neik et al., 2014) [42]	The study highlighted the current prevalence, diagnosis, treatment, and research on autism spectrum disorders (ASD) in Singapore and Malaysia.	A review paper from a different database.	--------------------	Based on database searches, it was found that awareness about autism among the lay and professional public is higher in Singapore compared to Malaysia.	
(Haimour and Obaidat 2013) [43]	This study endeavored to find out what school teachers knew about autism.	A total of 391 general and individual education teachers in Jeddah in Saudi Arabia.	Completed a study tool (autism knowledge questionnaire) to measure level of knowledge about autism.	It was found that among the participants, the knowledge about autism disorder ranged from satisfactory to almost weak.	

**Table 2:** Summary studies of beliefs about ASD.

Authors\years	Objective	Sample	Instrument	Result	Conclusion
(Samadi, 2020) [59]	Identification, description, and treatment of ASD in Iran.	A total of 43 Parents of children with ASD (27 mothers and 16 fathers).	Questionnaire.	The study result found Iranian parents had their special justification regarding their experience with ASD. Early child development and interventions must be understood within the cultural context.	The study suggested that the culturally informed researcher on ASD is vital to boost awareness of the importance of understanding parental concerns and their need for educational and psychological services in countries in which autism is less known, misdiagnosed, undiagnosed, or even stigmatized.
(Sheely, 2020) [60]	The aims of this study were to examine the context of the Indonesian government’s intention to develop an inclusive education system.	A total of 136 from teachers and educational therapists.	Questionnaire.	The data suggest that having access to information about autism in the Bahasa Indonesia language plays a role in educators’ beliefs about the stigmatization of teachers and parents of autistic children. Teachers’ epistemological beliefs were found to be linked to their beliefs in inclusive education.	
(Warstadt M., 2020) [61]	This study aimed to assess the public perceptions about autism spectrum disorder (ASD) among United States citizens by using Mechanical Turk.	The participants answered a survey about beliefs regarding causes, treatments, and general information of ASD.	Survey tool by online recruitment.	The results confirmed that participants who had a child with ASD were more likely to attribute ASD to external causes than those without connections to ASD.	The study’ result will support awareness campaigns.
(Stronach et al., 2019) [62]	The study aimed to explore autism understanding and stigma among university students, and general community members recruited at a state fair.	The result was that all the responses of ASD-Q fell within the adequate knowledge range, indicating relatively high levels of autism knowledge and low levels of stigma.	ASD-Q questionnaire.	The results of this study recommend the need for a continuous investigation into tools that indicate autism understanding and stigma.	
(Jegatheesan, et al., 2010) [54]	The study aimed to investigate the beliefs about autism among three multilingual immigrant South Asian Muslim families who have children with autism.	Parents.	Interviews and conversations recorded during 17 months.	The study’s results indicate that families have viewed that their primary purpose is to raise their child to incorporate them into daily social life, linguistic, and religious practices at home and in the community. On other words, Muslim families understand that the task of raising a child with autism in religious terms is the proper way to educate them.	
(Qi, 2016) [63]	This study tried to explain preschool teachers’ public beliefs about ASD.	A total of 215 Undergraduate university students in Macau.	Completed self-report measures assessing two beliefs concerning autism spectrum disorder etiology: (1) A belief in parental factors and (2) a belief in genetic factors.	The result confirmed that belief in ASD etiology statement is caused by negligent and emotional parenting, while one-third of participants believed in genetic etiology. However, participants expressed mild to moderate agreement with statements describing paternity as etiology in ASD.	
(Riany, 2016) [64]	The aim of this study was to examine how Indonesian mothers understand autism and the appropriate ways to parent such a child.	Nine Indonesian mothers	Using semi-structured interviews with nine Indonesian mothers.	The interviews revealed five related themes about autism, including traditional cultural beliefs about appropriate behavior during pregnancy, karma, and God’s plan, which is not usually reported in the literature from Western countries.	
(Hebert, 2010) [65].	This article is a review paper focused on parents’ beliefs about the cause and course of ASD.	The data were searched from 1995 to 2009; the keywords were autism, autistic disorder, belief, culture, parents, attitudes, and perceptions.	Review paper.	It was found in the review that parents hold a wide variety of beliefs about the cause of their child’s autism, including genetic factors, events surrounding the child’s birth, and environmental influences in the early childhood period. Some parents continue to attribute their child’s autism to immunizations, although more recent studies suggest the frequency may be decreasing. Some parents are pessimistic about their child’s future while others are hopeful that new strategies will be developed.	Some trust that society will become more accepting of their child’s idiosyncrasies. Parents’ beliefs about the cause of their child’s autism have been found to have an impact on decisions regarding future health care, family planning, and maternal mental health. The link between parental beliefs and their choices for interventions has not yet been empirically explored.
(Khanam R., 2018) [66]	The study aimed to increase awareness of society and participants of the family about autism.	Various respondents including parents, family members, neighbors, relatives, and therapists from Dhaka, Bogra, and Jessor district.	Open-ended questionnaire survey on various respondents.	The response was explanatory and analysis was done on the summary. It was observed that social negligence and lack of understanding have a greater impact on the development of autistic children, as well as increasing the suffering and insecurity of parents. Ideas have been provided based on the results of the analysis.	
(Bazzano, 2012) [67]	The study aimed to assess how parents change and discontinue their child’s vaccine schedule after their child is diagnosed with ASD, and assessment of how beliefs about the etiology of autism affect parents’ decision to do so.	A total of 197 eligible parents of children under 18 years of age.	Survey.	The result of this study found that parents changed vaccination practices and this change was associated with a belief that vaccines contributed to ASD.	The study suggests that educational tools should be designed to assist physicians when speaking to parents of children with ASD about vaccination.

**Table 3:** Summary studies of identification skills about ASD.

Authors\years	Objective	Sample	Instrument	Result	Conclusion
(Yasin M., 2020) [80]	This study aim is to identify teacher strategies and ability in identifying students with special needs.	Primary teachers.	This mixed method study involves 16 respondents in a qualitative study and 219 respondents in a quantitative study.	The study found that 50.2% of respondents achieve mastery level while 49.8% achieved less than mastery level. The study also found the ability to identify children with special education needs (SEN) based on their external behavior. Therefore, the qualitative study found that most of the teachers can identify children with disabilities through children’s behaviors and characteristics, while some of the respondents identify children based on academic performance, including children’s abilities to read and write.	
(Rosenbaum, 2019) [81]	The study aimed to understand pre-referral perception and decision factors involved.	Among 346 teachers.	[80]	The study found decision factors linked with play, social interactions, engagement, and verbal behaviors, but none were cited by a clear majority.	
(Splett J., 2019) [82]	The study examined the ability of teachers to accurately identify mental health concerns among elementary children.	A total of 153 teachers.	Vignette scenarios.	Findings indicated that teachers could accurately identify children with severe externalizing and internalizing problems. However, they were less accurate and less likely to think children with moderate or subclinical symptoms needed services.	
(Gabrielsen, 2019) [81]	The study aimed to better understand pre-referral perceptions and decision factors involved.	A total of 364 teachers and clinicians.	Multiple video clips from early signs of autism; the teachers and clinicians were asked to evaluate the child and to make decisions about ASD referral.	The result found that decision factors linked most often with play, social interaction, and verbal behaviors.	The study result confirmed the need for training in early childhood professionals; targeted training may encourage earlier referrals when autism is suspected in young children.
(Smith M., 2017) [83]	This study investigated whether teachers can recognize children’s anxiety and somatic symptoms, and how they identify children they perceive to be anxious or somatizing.	A sample of 1346 seven- to 11-year-old children, their 51 class teachers, and 144 parents took part in the study.	Data on children’s anxiety and somatic symptoms were collected using standardized scales and simple 1–5 teacher rating scales. Teachers were also asked to identify children they perceived to have “debilitating” levels of anxiety and (separately) somatic symptoms and to provide brief qualitative descriptions to explain their choices.	Small but significant positive associations were found between teachers’ and children’s reports of anxiety and somatic symptoms. Identified children reported similar levels of anxiety than children not identified, but significantly greater levels of somatic symptoms, although the size of this difference was modest. Teachers commonly described crying and avoidance as signs of anxiety.	Findings suggest that teachers show limited sensitivity to the variation in pupils’ levels of anxiety and somatic symptoms, and may struggle to identify children who may benefit from interventions or extra support in these domains.
(Deyessa A., 2017) [31]	The study examines teachers’ ability to identify children’s “debilitating” levels of anxiety and (separately) somatic symptoms and to provide brief qualitative descriptions to explain their choices.	Ethiopian teachers.	Data on children’s anxiety and somatic symptoms were collected using standardized scales and simple 1–5 teacher rating scales.	The result indicates that a teacher’s training was significantly associated with more accurate identification of a child.	
(Drusch, 2015) [8]	The study attempted to understand whether preschool teachers are familiar with signs of ASD in young children and their ability to discuss concerns with a child’s parents, and preschool teachers’ knowledge about diagnosis and intervention services in ASD.	Eighty-four preschool teachers.		The study result found preschool teachers have a moderate level of knowledge regarding ASD symptoms based on teachers’ experiences. Also, preschool teachers held positive perceptions about mainstreaming and those who have had training specific to inclusion.Teachers with greater experience reported comfort to express their concerns with a child’s parents.	Confirmed that professional or personal experience is not enough to increase a teacher’s knowledge and skills. In a similar vein, teachers cannot gain knowledge and skills in identifying and recognizing ASD just by working with children.

**Table 4:** Summary studies of self-efficacy about ASD.

Authors\years	Objective	Sample	Instrument	Result	Conclusion
(Sasson, 2018) [35]	To examine the effect of an early screening training on pediatric Physical Therapists PTs’: (1) Knowledge of autism spectrum disorder (ASD), (2) clinical self-efficacy, and (3) identification of markers.	Twenty-six pediatric PTs participated in a two-day ‘Early ASD Screening’ workshop.	Questionnaire in both ASD knowledge and self-Efficacy, and video case study.	The result confirmed that there is an increase in PTs’ knowledge and self-efficacy before and after the ASD workshop, and the PTs ability to identify the early signs of ASD is greater than before the workshop.	Conclude that the workshop is useful to increase the level of knowledge and self-efficacy among PTs.
(Drusch, 2015) [8]	The study attempted to understand whether preschool teachers are familiar with signs of ASD in young children and their ability to discuss concerns with a child’s parents, and preschool teachers’ knowledge about diagnosis and intervention services in ASD.	Eighty-four preschool teachers.		The study result found pre-school teachers have a moderate level of knowledge regarding ASD symptoms based on teachers’ experiences. Also, preschool teachers held positive perceptions about mainstreaming and those who have had training specific to inclusion. Teachers with greater experience-reported comfort to express their concerns with a child’s parents.	Confirmed that professional or personal experience is not enough to increase a teacher’s knowledge and skills. In a similar vein, teachers cannot gain knowledge and skills in identifying and recognizing ASD just by working with children.
(Arslan, 2017) [95]	This study investigates the effect of preschool teachers’ collective self-efficacy.	A study group consists of 172 preschool teachers who are working in public preschools affiliated with the Ministry of National Education in different cities of Turkey.	In this study, the teacher self- efficiency scale is employed to assess professional efficiency; it was found that there was a positive relationship between teachers’ self-efficacy and collective self-efficacy.	The study found that teachers’ self-efficacy can significantly explain collective self-efficacy.	Proficiency is the ability to have the professional knowledge, skills, and attitudes required to carry out tasks specific to a profession. In-service training activities for teachers will enable them to improve themselves in various subjects and providing training enabling them to benefit from their professional knowledge will contribute because, according to this research, the increase in the sense of occupational competence leads to the increase in collective self-efficacy levels.
(Gascoigne, M., 2019) [96]	This article aims to evaluate the efficacy of a brief in-service training workshop at increasing primary school teachers’ ADHD knowledge and sense of self-efficacy.	Teachers from 10 schools participated in the study (n = 274) and were allocated into either an intervention or waitlist control group. Teachers’ADHD knowledge and self-efficacy were assessed following the provision of a brief training workshop on ADHD. Knowledge and self-efficacy retention was also assessed at a one-month follow-up.		Results: Within the intervention group, ADHD knowledge and self-efficacy increased following the intervention. ADHD knowledge increased more than twofold, from very low to high levels, although increases in self-efficacy were more modest. Both knowledge and self-efficacy decreased at the one-month follow-up but, nevertheless, remained higher than baseline levels (*p* < 0.001).	Results demonstrate that a brief training workshop can increase primary school teachers’ ADHD knowledge.
